# Comparison of appropriate antimicrobial monotherapy and combination therapy in patients with carbapenem-resistant gram-negative bacilli bloodstream infections: a multicenter retrospective cohort study

**DOI:** 10.1128/spectrum.02559-25

**Published:** 2025-10-27

**Authors:** Ya-Li Sun, Yuan-Yuan Li, Rong Liufu, Shan Li, Tian-Yuan Zhu, Xian-Qing Shi, Xiao-Yun Fu, Yong Liu, Feng Shen, Xiang Zhou, Li Weng, Jin-Min Peng, Bin Du

**Affiliations:** 1 Medical ICU, State Key Laboratory of Complex Severe and Rare Diseases, Peking Union Medical College Hospital, Peking Union Medical College and Chinese Academy of Medical Sciences, Beijing, China; 2 Department of Critical Care Medicine, Shandong Provincial Hospital Affiliated to Shandong First Medical University 34708 , Jinan, China; 3 Department of Critical Care Medicine, Guizhou Provincial People’s Hospital, Guiyang, China; 4 Department of Critical Care Medicine, Affiliated Hospital of Zunyi Medical University 159358 , Zunyi, China; 5 Department of surgery ICU, ZhangZhou Hospital of Fujian Province, Zhangzhou, China; 6 Department of Critical Care Medicine, The Affiliated Hospital of Guizhou Medical University 74720, Guiyang, China; 7 Department of Critical Care Medicine, Information Center, State Key Laboratory of Complex Severe and Rare Diseases, Peking Union Medical College Hospital, Peking Union Medical College and Chinese Academy of Medical Sciences, Beijing, China; Universita degli Studi dell'Insubria, Varese, Italy

**Keywords:** carbapenem-resistant gram-negative bacilli, bloodstream infections, combination therapy, monotherapy, clinical outcomes

## Abstract

Whether appropriate monotherapy or combination therapy is more effective for carbapenem-resistant gram-negative bacilli bloodstream infections (CRGNB-BSI) is still a matter of debate. This multicenter retrospective study included CRGNB-BSI patients who received either appropriate monotherapy (only one active agent) or combination therapy (at least two active agents). The primary outcome was 28-day all-cause mortality, while the secondary outcome was 14-day clinical cure. Inverse probability of treatment weighting (IPTW) was used to adjust confounding factors for the choice of therapeutic strategies. Multivariable regression analysis was performed in the IPTW cohort. Of 237 patients included, 155 received appropriate monotherapy and 82 received combination therapy. The overall 28-day mortality was 40% (94/237), with no significant difference between the combination group (37%, 30/82) and the monotherapy group (41%, 64/155; *P* = 0.479). In multivariable analysis of the IPTW cohort, combination therapy was not associated with reduced 28-day mortality (aHR 0.65; 95% CI, 0.40–1.07; *P* = 0.088) or increased 14-day clinical cure rate (aOR 1.04; 95% CI, 0.71–1.51; *P* = 0.855). Subgroup analyses demonstrated significant 28-day survival benefits of combination therapy among patients who received early appropriate treatment (started within 48 hours after infection), those with septic shock, and those treated with regimens including tetracycline or polymyxin B (*P* < 0.01). In patients with CRGNB-BSI, appropriate combination therapy was not associated with improved overall survival compared to monotherapy. However, it may offer survival benefits when initiated early, in patients with septic shock, and when regimens include tetracyclines or polymyxin B.

**IMPORTANCE:**

Carbapenem-resistant gram-negative bacilli bloodstream infections remain a critical therapeutic challenge with high mortality and few effective options. In a real-world, multicenter cohort using inverse probability weighting to control for confounding, we found no overall benefit of appropriate combination therapy versus monotherapy in survival or early clinical cure. However, combination therapy was associated with improved 28-day survival in specific contexts: when initiated within 48 hours, in patients with septic shock, and when regimens included tetracyclines or polymyxin B. These findings highlight the importance of timely, targeted combination therapy for selected high-risk patients, while supporting judicious monotherapy use otherwise.

## INTRODUCTION

Carbapenem resistance has emerged as a critical global health challenge and a major contributor to mortality (1). Carbapenem-resistant gram-negative bacilli (CRGNB), including *Acinetobacter baumannii* (CRAB), *Enterobacterales* (CRE), and *Pseudomonas aeruginosa* (CRPA), have been classified as “critical” or “high-priority” pathogens by the World Health Organization due to their significant health burden and limited treatment options (2). Among CRGNB-related infections, bloodstream infections (BSIs) are life-threatening invasive manifestations with mortality rates of 21% to 73% (3–7), highlighting the urgent need for evidence-based treatment strategies in the context of limited antimicrobial options.

Combination antimicrobial therapy is often employed in clinical practice for severe infections to enhance bacterial eradication and reduce the development of resistance (8–11). BSI, as a severe type of infection, is among the most common scenarios (12). However, the benefit of combination therapy over monotherapy for CRGNB infections remains controversial. While some retrospective studies and meta-analyses suggested that combination therapy offered survival advantages, especially in certain high-risk subgroups or pathogen species (13–15), prospective studies have failed to show definite benefits (16–18). Current guidance regarding combination therapy versus monotherapy for CRGNB infections differs according to the specific pathogens and lacks comprehensive, evidence-based recommendations tailored to different infection sites, such as BSI (19, 20). In addition, in settings where novel antibiotics are not readily available and many patients have complicated conditions, clinicians are often forced to use alternative regimens. Therefore, more real-world research is needed to assess the efficacy of various antimicrobial strategies for CRGNB-BSIs.

In this study, we aimed to compare the effects of appropriate antimicrobial monotherapy versus combination therapy on 28-day all-cause mortality and 14-day clinical cure rates among hospitalized patients with CRGNB-BSIs. Additionally, subgroup analyses were performed by infection source, bacterial species, disease severity, antimicrobial regimens, and timing of therapy.

## MATERIALS AND METHODS

### Study design and population

This retrospective, multicenter cohort study was conducted between January 2018 and March 2023 at five tertiary hospitals in China. Adult inpatients diagnosed with CRGNB-BSI who received appropriate antimicrobial therapy were included, with only the first episode considered for each patient. Patients were excluded if they had a blood co-culture positive for non-CRGNB pathogens, lacked sufficient clinical data, received appropriate antimicrobial therapy for less than 48 hours, or died within 48 hours from the onset of CRGNB-BSI. Patients were categorized into monotherapy and combination therapy groups according to their antimicrobial regimens. The primary outcome was 28-day all-cause mortality after the onset of CRGNB-BSI, and the secondary outcome was 14-day clinical cure rate. Patients discharged within 28 days of the CRGNB-BSI onset were contacted by telephone to collect information about the outcomes.

This study was approved by the Ethics Committee of Peking Union Medical College Hospital (K23C3906). Given the retrospective nature of the study, the requirement for written informed consent was waived.

### Definitions

According to the 2018 CLSI breakpoint standards, we defined CRGNB as gram-negative bacteria that exhibit resistance or intermediate susceptibility to any carbapenem antibiotic (21). BSI was defined as the isolation of a pathogen from one or more blood cultures obtained from a patient with suspected blood infection. The onset of CRGNB-BSI was defined as the date of the first positive CRGNB blood culture collection. Hospital-acquired CRGNB-BSI was defined as a positive blood culture obtained ≥48 hours after hospital admission, while ICU-acquired CRGNB-BSI was defined as a positive blood culture obtained more than 48 hours after ICU stay.

Appropriate antimicrobial therapy was defined as administration of at least one *in vitro* active antimicrobial agent against the bloodstream isolates within 5 days from the CRGNB-BSI onset and continued for at least 48 hours. Early therapy was defined as initiation of appropriate antimicrobials within 48 hours of CRGNB-BSI onset (13). Monotherapy was defined as treatment with a single appropriate antimicrobial, while combination therapy referred to concurrent administration of at least two appropriate antimicrobials. Given that the study period (2018–2023) coincided with several updates of guidelines for CRAB, CRE, and CRPA treatment, adherence to antibiotic recommendations was assessed according to the first edition of the IDSA guidance for CRGNB infections (22, 23).

Clinical cure was defined as the complete resolution of clinical symptoms and signs associated with CRGNB-BSI, such as normalization of body temperature, heart rate <100 beats per minute, and normalization of white blood cell count (24, 25). Patients discharged alive from the ICU within 14 days of CRGNB-BSI onset were also considered clinically cured.

### Data collection

The following data were extracted from hospitals’ electronic medical record systems: demographic characteristics, comorbidities, clinical and laboratory findings, microbiological data (infection source, bacterial species, and antimicrobial susceptibility results), as well as information on disease severity, including the Pitt bacteremia score, ICU admission, and organ support information at the onset of CRGNB-BSI; antimicrobial therapy regimens; and outcomes, including length of hospital stay, 28-day mortality, and 14-day clinical cure rate.

### Statistical analysis

Descriptive statistics were reported as median (interquartile range, IQR) for continuous variables and as counts (percentage) for categorical variables. Intergroup differences were compared using the Wilcoxon rank sum test for continuous variables, and χ test or Fisher’s exact test, as appropriate, for categorical variables, respectively.

Inverse probability of treatment weighting (IPTW) was used to adjust confounding factors for the choice of therapeutic strategies by assigning different weights to individuals, thereby allowing the study to simulate the effects of randomized controlled trials. Firstly, propensity scores (PS), representing the conditional probability of receiving combination therapy, were estimated through logistic regression. Variables included in the model were those with statistically significant intergroup differences or those considered clinically relevant to the use of combination therapy. The weights of IPTW were calculated as the inverse of the PS for the combination therapy group and the inverse of (1 - PS) for the monotherapy group (9). Standardized mean differences (SMD) before and after IPTW adjustment are presented in Supplementary Figure, with SMD <0.1 indicating adequate balance between groups. Subsequent analyses were all conducted based on the IPTW cohort.

Overall survival was evaluated by Kaplan–Meier analysis from the onset of CRGNB-BSI onset to 28 days, with group comparisons performed using the log-rank test. Multivariable Cox and logistic regression analyses were performed on the IPTW cohort to evaluate the association between combination therapy and 28-day mortality and 14-day clinical cure, respectively. Variables associated with 28-day mortality in the univariate analysis (*P* < 0.05) and those with clinical significance were included in the multivariable models. Covariates already incorporated into the IPTW model and those highly correlated were excluded from the multivariable analysis to avoid multicollinearity. Subgroup analyses were also conducted based on infection source, bacterial species, disease severity, antimicrobial regimens, and timing of therapy.

All statistical analyses were performed using R version 4.4.2. A two-sided *P* < 0.05 was considered statistically significant.

## RESULTS

**Fig 1:**
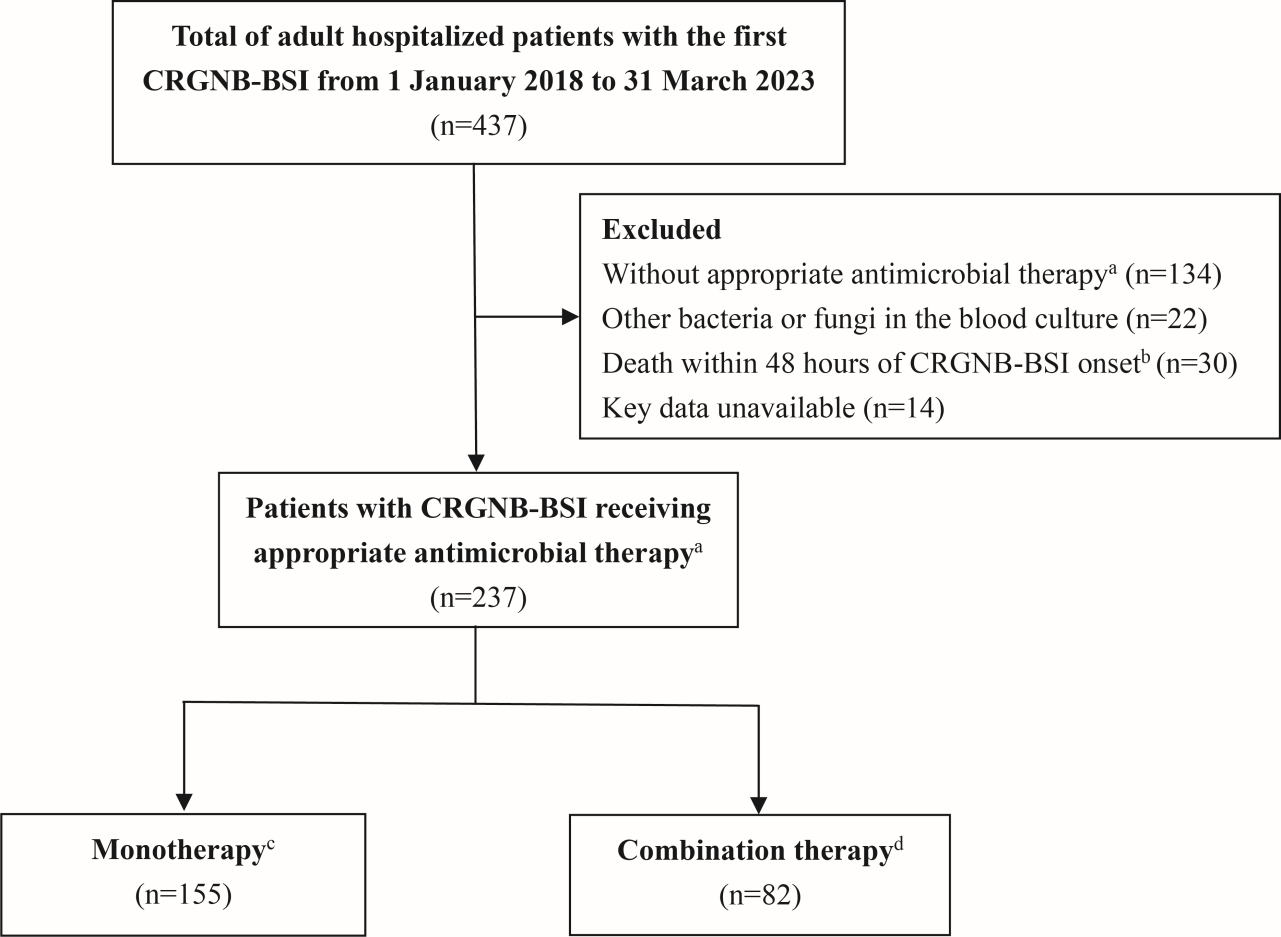
Flow chart of included patients. Abbreviations: CRGNB-BSI, carbapenem-resistant gram-negative bacilli bloodstream infections. ^a^Appropriate therapy was defined as administration of at least one *in vitro* active antimicrobials against the isolates within 5 days from the CRGNB-BSI onset and for at least 48 hours. ^b^The onset of CRGNB-BSI was defined as the day of positive CRGNB blood culture collection. ^c^Antimicrobial monotherapy was defined as treatment with a single appropriate antimicrobial. ^d^Antimicrobial combination therapy was defined as the concurrent administration of at least two appropriate antimicrobials.

During the study period, 437 adult hospitalized patients with first-episode CRGNB-BSI were identified, among whom 237 met the inclusion criteria (Fig. 1). The median age was 63 years (interquartile range [IQR], 50–72), and 68% were male. A total of 107 patients presented with shock at the onset of CRGNB-BSI, and 132 patients were admitted to the ICU. Among these patients, 155 were treated with appropriate monotherapy, and 82 received combination therapy. Baseline characteristics of the study population receiving combination therapy versus monotherapy are presented in Table 1. There were no significant differences between groups regarding the rate of shock, requirement for invasive mechanical ventilation (IMV), or time to blood culture positivity. Patients with immunodeficiency (62% vs 48%, *P* = 0.038), CRE-BSI (44% vs 30%, *P* = 0.032), pneumonia source (59% vs 42%, *P* = 0.010), or hospital-acquired BSI (89% vs 78%, *P* = 0.032) were more likely to receive combination therapy. After IPTW adjustment, the difference in variables between groups was well balanced (Fig. S1).

**TABLE 1:** Comparison between patients treated with monotherapy or combination therapy^*a*^^,^^*b*^

Variables	Monotherapy (*n* = 155)	Combination therapy (*n* = 82)	*P*-value
Age (years)	63 (52, 73)	59 (44, 70)	0.155
Male sex	106 (68%)	56 (68%)	0.988
CCI score (points)	5 (4, 7)	5 (3, 7)	0.724
Comorbidities diseases			
Diabetes mellitus	49 (32%)	25 (30%)	0.858
COPD	7 (4.5%)	0 (0%)	0.116
CKD receiving dialysis	8 (5.2%)	4 (4.9%)	0.827
Congestive heart failure	21 (14%)	8 (9.8%)	0.394
Immuno-deficiency	75 (48%)	51 (62%)	0.038
Source of infection			
Pulmonary	65 (42%)	48 (59%)	0.010
Abdominal	41 (26%)	13 (16%)	0.059
Catheter-related	5 (3.2%)	1 (1.2%)	0.615
Other sites	24 (15%)	10 (12%)	0.489
Unknown	20 (13%)	10 (12%)	0.875
Hospital-acquired	121 (78%)	73 (89%)	0.032
ICU-acquired	71 (46%)	36 (44%)	0.778
Microbiologic data			
Blood culture time to positivity (hours)	14 (12, 17)	14 (10, 16)	0.337
Type of bacteria			
CRAB	48 (31%)	19 (23%)	0.201
CRE	47 (30%)	36 (44%)	0.032
CRPA	35 (23%)	21 (26%)	0.559
Other pathogens	25 (16%)	6 (7.3%)	0.017
Laboratory data at CRGNB-BSI onset			
White blood cell count (×10^9^ /L)	10.0 (5.13, 15.9)	10.5 (3.31, 14.6)	0.460
Neutrophil counts (×10^9^ /L)	8.65 (3.85, 14.1)	8.19 (2.78, 12.7)	0.479
Lymphocyte counts (×10^9^ /L)	0.62 (0.33, 0.93)	0.42 (0.21, 0.97)	0.065
Platelet counts (×10^9^ /L)	99 (48, 179)	90 (35, 190)	0.952
Alanine aminotransferase (U/L)	36.0 (17.5, 62.9)	29.0 (15.3, 73.3)	0.523
Creatinine (μmol/L)	82.5 (54.0, 158)	82.2 (61.0, 140)	0.773
Disease severity			
ICU admission	84 (54%)	48 (59%)	0.520
Pitt score	3 (1, 5)	3 (1, 5)	0.575
Shock	70 (45%)	37 (45%)	0.995
IMV	81 (52%)	44 (54%)	0.834
CRRT	29 (19%)	20 (24%)	0.301
Early therapy^*c*^	93 (60%)	51 (62%)	0.741
Duration of appropriate therapy (days)	9 (5, 14)	11 (7, 15)	0.009
Outcomes			
28-day all cause mortality	64 (41%)	30 (37%)	0.479
14-day all cause mortality	49 (32%)	23 (28%)	0.568
14-day clinical cure	84 (54%)	46 (56%)	0.778
Hospital LOS (days)	22 (10, 36)	30 (12, 50)	0.041

^*a*^ Data are presented as number (percentage) or median (interquartile range).

^*b*^ CRGNB-BSI, carbapenem-resistant Gram-negative bacilli bloodstream infections; CCI, Charlson comorbidity index; COPD, chronic obstructive pulmonary disease; ICU, intensive care unit; CKD, chronic kidney disease; IMV, invasive mechanical ventilation; CRRT, continuous renal replacement therapy; CRAB, carbapenem-resistant *Acinetobacter baumannii*; CRE, carbapenem-resistant *Enterobacterales*; CRPA, carbapenem-resistant *Pseudomonas aeruginosa*; LOS, length of stay.

^*c*^ Early therapy was defined as initiation of appropriate antimicrobials within 48 hours of CRGNB-BSI onset.

The detailed antimicrobial regimens are presented in Table 2. In the monotherapy group, tetracyclines were the most frequently administered antimicrobials (56/155, 36%), followed by β-lactam/β-lactamase inhibitors (BL-BLI, 38/155, 25%). In combination therapy group, the most common regimen was polymyxin B (PMB) plus tetracycline (32/82, 39%). All patients received appropriate antibiotics, yet only 70 (29.5%) received regimens that were adherent to antibiotic recommendations according to the first edition of the IDSA guidance for CRGNB infections (22, 23).

**TABLE 2:** Appropriate antimicrobial regimens among overall population^*a*^

	CRE (*n* = 83)	CRAB (*n* = 67)	CRPA (*n* = 56)	Other pathogens (*n* = 31)
Monotherapy	47 (57%)	48 (72%)	35 (63%)	25 (81%)
Polymyxin B	4 (5%)	11 (16%)	6 (11%)	0
Tetracycline	21 (25%)	33 (49%)	0	2 (6%)
BL-BLI^*b*^	8 (10%)	4 (6%)	18 (32%)	9 (29%)
Other^*c*^	14 (17%)	0	11 (20%)	14 (45%)
Combination therapy	36 (43%)	19 (28%)	21 (37%)	6 (19%)
Polymyxin B + tetracycline	16 (19%)	16 (24%)	0	0
Polymyxin B + BL-BLI^*b*^	1 (1%)	1 (1%)	5 (9%)	0
Polymyxin B + other^*c*^	1 (1%)	0	1 (2%)	0
Tetracycline + BL-BLI^*b*^	6 (7%)	2 (3%)	0	2 (6%)
Tetracycline + other^*c*^	9 (11%)	0	0	1 (3%)
BL-BLI + other^*c*^	0	0	8 (14%)	1 (3%)
Others^*c*^	3 (4%)	0	7 (12%)	2 (6%)

^*a*^ Data are presented as *n* (%).

^*b*^ BL-BLI: β-Lactams/β-lactamase inhibitors, including ceftazidime avibactam.

^*c*^ Other: Antimicrobials except polymyxin B, tetracycline, or BL-BLI.

**Fig 2:**
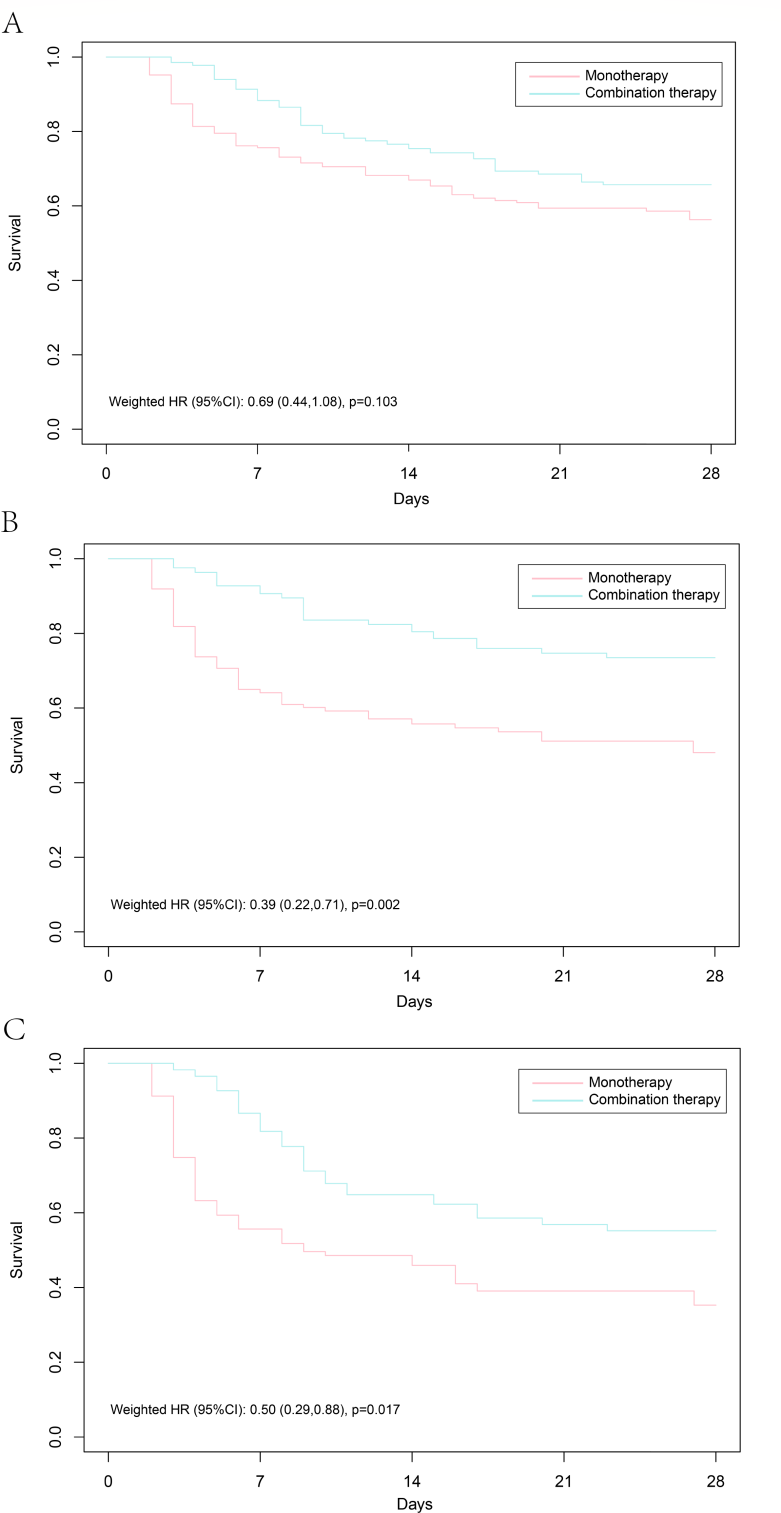
28-day cumulative survival in patients treated with monotherapy versus combination therapy in IPTW cohort. (**A**) All population, (**B**) early therapy population, and (**C**) shock population. Abbreviations: IPTW, inverse probability of treatment weighting; HR, hazard ratio; CI, confidence interval.

**Fig 3:**
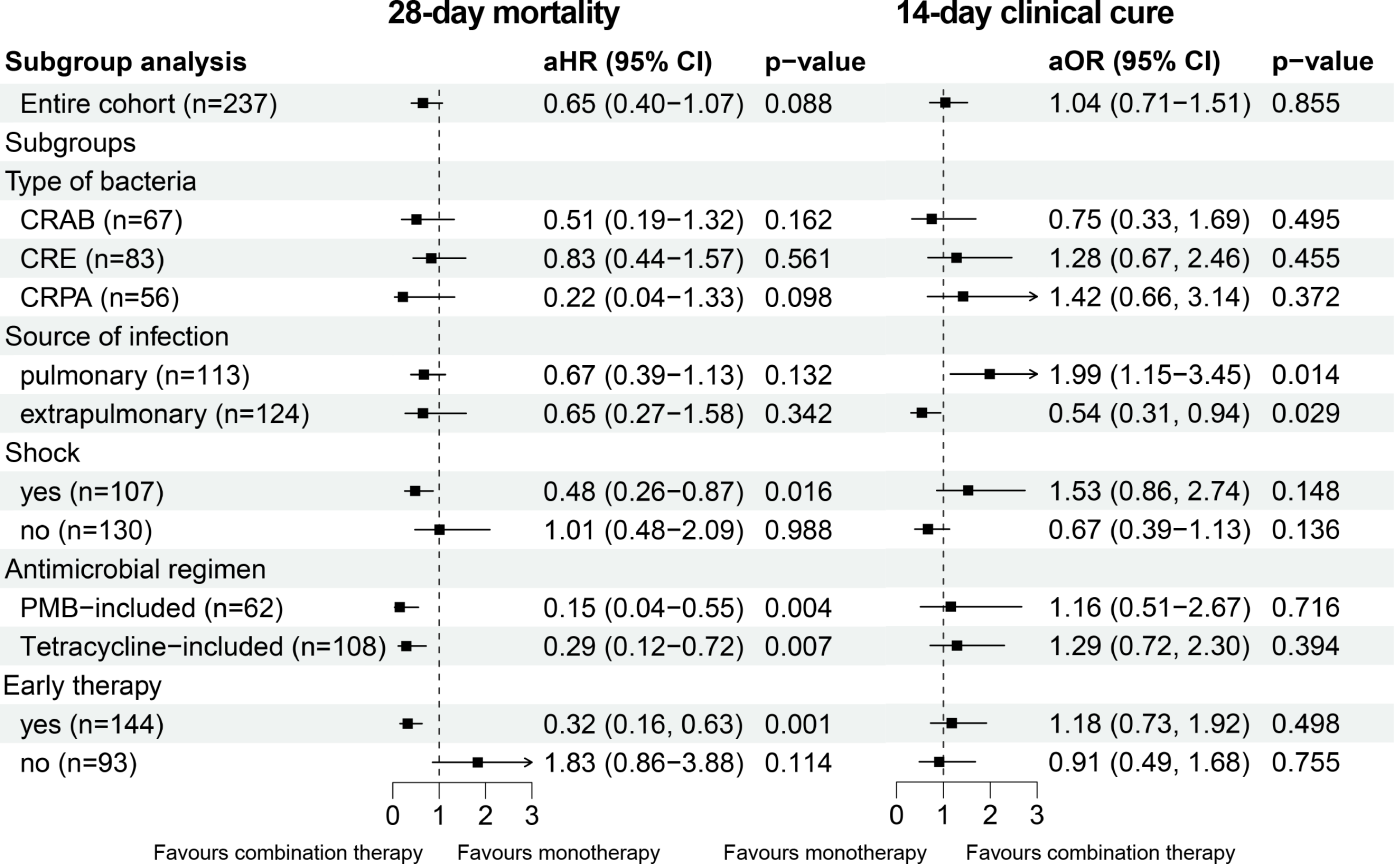
Association between combination therapy and study outcomes: results of multivariable Cox/logistic regression analyses in the entire cohort and subgroups of IPTW-adjusted population. The multivariable models were adjusted on Pitt score, lymphocyte counts, and platelet counts. For antibiotic therapy based on tetracycline, CRPA-BSI patients were excluded from the analysis population. Abbreviations: PMB, polymyxin B.

The 28-day mortality rate for the overall cohort was 39.7% (94/237), with 41.3% (64/155) in the monotherapy group and 36.6% (30/82) in the combination therapy group (*P* = 0.479). In the IPTW-adjusted cohort, 28-day survival remained comparable between the two groups (HR 0.69; 95% CI, 0.44–1.08; *P* = 0.103) (Fig. 2). In univariate analysis, Charlson Comorbidity Index (CCI) scores, immunodeficiency status, lymphocyte and platelet counts, infection sources, bacterial species, and disease severity were associated with 28-day mortality (Table S1). Further multivariable Cox regression analysis adjusting for these variables in the IPTW-adjusted cohort also demonstrated no statistically significant benefit of combination therapy for 28-day mortality (aHR 0.65; 95% CI, 0.40–1.07; *P* = 0.088) (Fig. 3). Moreover, although less than 30% of patients in this cohort received antimicrobial regimens concordant with the first edition of the IDSA guidance, such adherence was not associated with improved 28-day survival (aHR 0.85; 95% CI, 0.51–1.41; *P* = 0.527). As for the secondary outcome, the 14-day clinical cure rates were similar between the two groups, with 54% (84 of 155) in the monotherapy versus 56% (46 of 82) in the combination therapy group. Multivariable logistic regression analysis showed no statistically significant difference as well (aOR 1.04; 95% CI, 0.71–1.51; *P* = 0.855) (Fig. 3).

Figure 3 presents the results of subgroup analyses for 28-day mortality and 14-day clinical cure. Notably, combination therapy was significantly associated with reduced 28-day mortality in several subgroups, including patients with shock (aHR 0.48; 95% CI, 0.26–0.87; *P* = 0.016), those who received early therapy (aHR 0.32; 95% CI, 0.16–0.63; *P* = 0.001), and patients whose regimens included PMB (aHR 0.15; 95% CI, 0.04–0.55; *P* = 0.001) or tetracyclines (aHR 0.29; 95% CI, 0.12–0.72; *P* = 0.001). However, combination therapy in these subgroups did not demonstrate a significant advantage in terms of the 14-day clinical cure. Additionally, no association between combination therapy and improved outcomes was observed in either the different pathogen subgroups or the pulmonary and non-pulmonary source subgroups.

## DISCUSSION

In this retrospective study, we demonstrated that appropriate combination antimicrobial therapy did not significantly improve 28-day mortality or 14-day clinical cure compared to appropriate monotherapy in patients with CRGNB-BSI. However, combination therapy appeared to provide survival benefits among patients with septic shock, those receiving early treatment, or when regimens include tetracyclines or PMB.

Although antimicrobial management of CRGNB-BSI remains challenging, comparative data on monotherapy versus combination therapy are scarce. Our findings are consistent with previous studies, including randomized controlled trials (RCTs) and observational studies, which have not demonstrated a significant benefit of combination therapy. In one RCT including 406 patients with severe CRGNB infections, nearly half of whom had BSIs, there was no significant difference in 14-day clinical failure between PMB monotherapy and combination therapy. However, the combination regimen evaluated in that RCT was PMB plus meropenem, which is no longer recommended for CRGNB infections, thus limiting its relevance to current clinical practice (17). Two retrospective studies likewise did not show a survival benefit with combination therapy (26, 27), but neither balanced baseline characteristics nor restricted analyses to appropriate antimicrobial regimens, potentially masking any benefits of appropriate combination therapy. This matters because appropriate antimicrobial therapy and unbiased allocation of treatment strategies are prerequisites for valid comparisons across regimens (3, 28). Notably, one of these studies, which was also conducted in China, did not include PMB and ceftazidime-avibactam, as these agents were unavailable during the study period (2012–2017) in China (26). In contrast, our analysis focused exclusively on appropriate antimicrobials and incorporated antimicrobial regimens more closely aligned with contemporary clinical practice, thereby enhancing clinical relevance.

Although combination therapy showed no overall benefit in the cohort, potential advantages were identified in subgroup analyses in certain populations, particularly among those receiving early treatment and those with septic shock. These findings underscored the importance of timely initiation of combination therapy and identification of appropriate patients. The potential benefits of early combination therapy were likely attributable to a high bacterial clearance in the early stages of infection. However, as the initiation of treatment was delayed, the advantages of appropriate combination therapy may diminish. Previous studies have consistently shown that appropriate antibiotic therapy should be initiated as early as possible for optimal outcomes (29–31). Our study further highlighted the significance of the “time window effect,” suggesting that the benefits of combination therapy are most evident when administered early in the course of infection. Furthermore, in order to achieve early appropriate therapy, which means selecting suitable antimicrobials before blood culture results are available, it is necessary to improve the prediction of CRGNB infections by integration of validated, high-performance clinical prediction models with rapid carbapenemase detection assays. In addition, we found that combination therapy was associated with lower mortality in patients with septic shock, which was consistent with previous studies (13, 32). In the study by Marco Falcone and colleagues, the association between combination therapy and reduced mortality was observed only in patients with septic shock or severe chronic conditions in CRKP-BSI (32), suggesting that combination therapy may provide greater benefits in patients with severe infections. Nevertheless, these findings should be interpreted with caution due to the inherent limitations of subgroup analyses. Further research is needed to explore the potential benefits of combination therapy in critically ill patients, such as those with septic shock.

The efficacy of monotherapy versus combination therapy may vary depending on the specific antimicrobials. In tetracycline-treated CRGNB-BSI, our subgroup analyses demonstrated lower 28-day mortality with combination therapy (41%) than monotherapy (57%). In line with this finding, prior studies showed relatively low in-hospital mortality and higher clearance of CRE infection when tetracyclines were used in combination rather than alone (33, 34). Tetracyclines, particularly tigecycline, have a large volume of distribution and achieve relatively low serum concentrations. Consistent with this pharmacokinetic limitation, evidence indicates that tigecycline monotherapy yielded worse outcomes in ventilator-associated pneumonia, particularly those with bacteremia at baseline (35). Accordingly, current guidelines do not recommend tetracyclines for BSI (20). Nevertheless, tetracyclines were frequently used in our cohort. This likely reflected real-world constraints, including limited access to newer agents, a substantial proportion of patients with renal dysfunction, and the presence of metallo-β-lactamase–producing isolates, which curtailed other treatment options. Real-world deviations from guideline-concordant therapy are common under such circumstances (13, 36, 37). These mechanistic and our study support the rationale that when tetracyclines have to be used for CRGNB-BSIs, combination therapy should be preferable to monotherapy. Similarly, among patients treated with PMB, lower mortality was associated with combination therapy compared to monotherapy. Monotherapy performs poorly, perhaps in part because dosing of PMB may not have been adequate in some patients, particularly in infections such as bacteremia that require high plasma concentrations. Given that PK/PD studies of PMB for optimal probability of target attainment had not been as extensive as those conducted for many other antibiotics, an international consensus guideline was published in 2019 (38). However, standardized PMB dosing and therapeutic drug monitoring (TDM) were not widely implemented during our study period, and TDM remains limited in many centers even now. Therefore, our findings support the use of combination therapy when treating CRGNB-BSIs with PMB in settings where standardized PMB dosing and TDM cannot be performed. Future research could consider monitoring the PK/PD of PMB to better clarify the efficacy of monotherapy and combination therapy.

This study has several limitations. Although we used IPTW to reduce confounding bias, residual unmeasured confounding may persist. In addition, deviations from international guidances, such as the high rate of tetracycline use, may have influenced the assessment of treatment effects. However, our findings remain informative and reflect real-world practice in settings with limited access to newer agents. Nevertheless, these findings should be interpreted cautiously and require external validation. Furthermore, combination therapy showed a favorable but non-significant association with reduced 28-day mortality in the entire cohort, reflecting limited statistical power. Thus, further larger, adequately powered studies were warranted to confirm this potential benefit. Finally, we did not evaluate drug toxicity or treatment costs associated with combination therapy, both of which may impact clinical decision-making.

Taken together, our data indicated that among patients with CRGNB-BSI, appropriate antimicrobial combination therapy had no significant survival advantage over monotherapy, but may benefit patients with shock and those receiving early antimicrobial therapy. Moreover, when the use of tetracycline or PMB is unavoidable in clinical practice, our findings suggested that combination therapy should be preferred over monotherapy.

## Data Availability

The datasets used and/or analyzed in the present study are available from the corresponding author on reasonable request.

## SUPPLEMENTAL MATERIAL

Supplemental Material spectrum.02559-25-s0001.pdf Supplemental figure and table.

ASM does not own the copyrights to Supplemental Material that may be linked to, or accessed through, an article. The authors have granted ASM a non-exclusive, world-wide license to publish the Supplemental Material files. Please contact the corresponding author directly for reuse.
