# Supplementary material for: Comparison of appropriate antimicrobial monotherapy and combination therapy in patients with carbapenem-resistant gram-negative bacilli bloodstream infections: a multicenter retrospective cohort study
Source: Microbiol Spectr. 2025 Oct 27;13(12):e02559-25. doi: 10.1128/spectrum.02559-25 (PMC12671182; doi:10.1128/spectrum.02559-25)
Supplement: Supplemental Material — Supplemental figure and table. [file spectrum.02559-25-s0001.pdf]

Supplementary Figure. Standardized mean difference (SMD) before and after IPTW adjustment. Abbreviations: IPTW, inverse probability of treatment weighting.

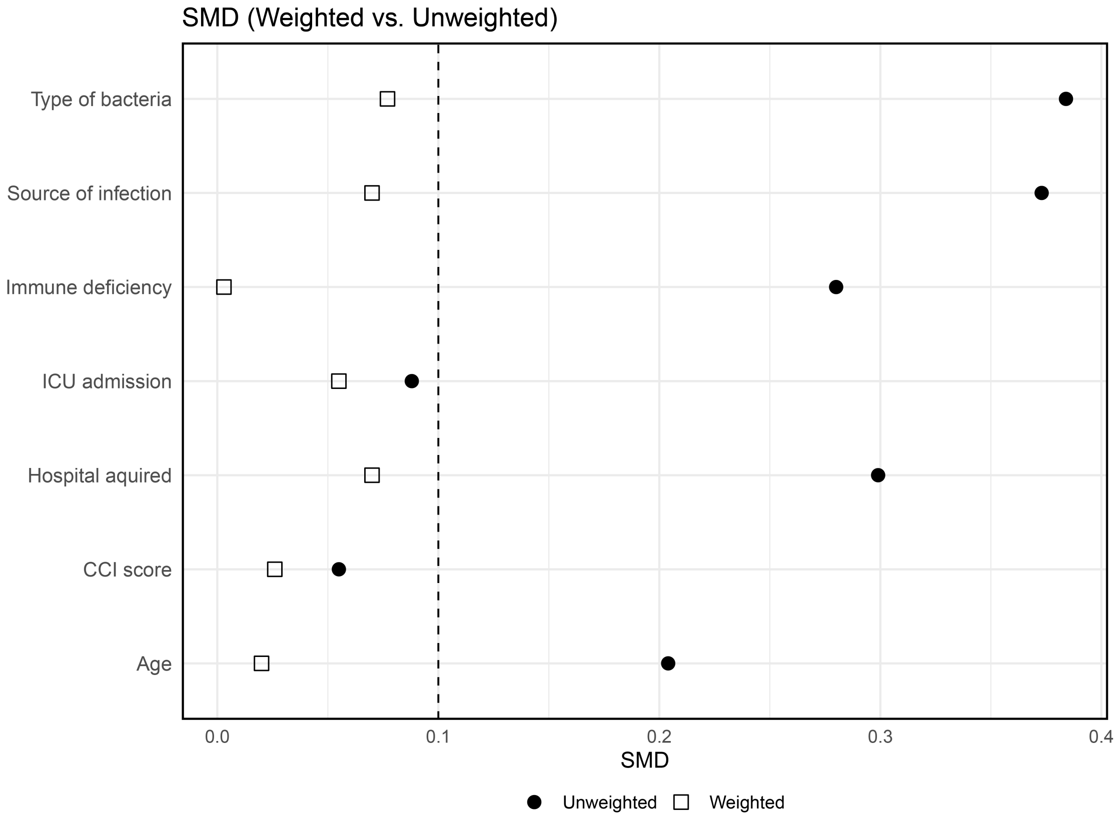

Supplementary Table. Univariate analysis comparing survivors and non-survivors at 28-days from CRGNB-BSI onset.

	**Survivors** (n=143)	**Non-survivors** (n=94)	**p-value**
Age (years)	61 (48, 72)	64 (52, 73)	0.460
Sex (male)	100 (70%)	62 (66%)	0.518
CCI score (points)	5 (3, 7)	6 (4, 7)	0.008
Comorbidities diseases			
Diabetes mellitus	40 (28%)	34 (36%)	0.178
COPD	3 (2.1%)	4 (4.3%)	0.568
CKD receiving dialysis	7 (4.9%)	5 (5.3%)	0.875
Congestive heart failure	17 (12%)	12 (13%)	0.839
Immuno-deficiency	68 (48%)	58 (62%)	0.028
Source of infection			
Pneumonia	55 (38%)	58 (62%)	0.001
Abdominal	39 (27%)	15 (16%)	0.037
Catheter-related	3 (2.1%)	3 (3.2%)	0.919
Other sites	23 (16%)	11 (12%)	0.343
Unknown	23 (16%)	7 (7.4%)	0.045
Hospital-acquired	108 (76%)	86 (91%)	0.001
ICU-acquired	48 (34%)	59 (63%)	0.001
Microbiologic data			
Blood culture time to positivity (hours)	15 (12, 17)	13 (11, 16)	0.116
Type of bacteria			
CRAB	29 (20%)	38 (40%)	0.001
CRE	47 (33%)	36 (38%)	0.388
CRPA	45 (31%)	11 (12%)	0.001
Other pathogens	22 (15%)	9 (9.6%)	0.190
Laboratory data at CRGNB-BSI onset			

	**Survivors** (n=143)	**Non-survivors** (n=94)	**p-value**
White blood cell counts (×10^9/L)	10.1 (4.79, 15.6)	10.5 (5.29, 16.9)	0.513
Neutrophil counts (×10^9/L)	7.97 (3.66, 13.4)	9.36 (4.36, 14.4)	0.327
Lymphocyte counts (×10^9/L)	0.67 (0.38, 1.02)	0.38 (0.20, 0.73)	0.001
Platelet counts (×10^9/L)	130 (62, 206)	72 (34, 143)	0.001
Alanine aminotransferase (U/L)	31.6 (16.6, 56.5)	37.0 (13.0, 79.0)	0.930
Creatinine (μmol/L)	79.0 (54.5, 128)	94.4 (61.3, 173)	0.149
Disease severity			
ICU admission	66 (46%)	66 (70%)	0.001
Pitt bacteraemia score (points)	2 (1, 4)	4 (2, 6)	0.001
Shock	45 (31%)	62 (66%)	0.001
IMV	58 (41%)	67 (71%)	0.001
CRRT	27 (19%)	22 (23%)	0.397
Combination therapy	52 (36%)	30 (32%)	0.479
Early therapy[a]	84 (59%)	60 (64%)	0.430

Data are presented as number (percentage) or median (interquartile range).

Abbreviations: CRGNB-BSI, carbapenem-resistant Gram-negative bacilli bloodstream infections; CCI, Charlson comorbidity index; COPD, chronic obstructive pulmonary disease; ICU, intensive care unit; CKD, chronic kidney disease; IMV, invasive mechanical ventilation; CRRT, continuous renal replacement therapy; CRAB, carbapenem-resistant *Acinetobacter baumannii*; CRE, carbapenem-resistant *Enterobacterales*; CRPA, carbapenem-resistant *Pseudomonas aeruginosa*.

[a]Early therapy was defined as initiation of appropriate antimicrobials within 48 hours of CRGNB-BSI onset.
